# Evaluation of hospital pharmacists’ activities using additional reimbursement for infection prevention as an indicator in small and medium-sized hospitals

**DOI:** 10.1186/s40780-023-00327-5

**Published:** 2024-01-10

**Authors:** Yuichi Tasaka, Takeshi Uchikura, Shiro Hatakeyama, Daisuke Kikuchi, Masami Tsuchiya, Ryohkan Funakoshi, Taku Obara

**Affiliations:** 1 Laboratory of Clinical Pharmacy, School of Pharmacy, Shujitsu University, 1-6-1 Nishigawara, Naka-ku, Okayama, Okayama 703-8516 Japan; 2 First Subcommittee, Committee on Academic, The Japanese Society of Hospital Pharmacists, 2-12-15, Shibuya, Shibuya-ku, Tokyo, 150-0002 Japan; 3 Department of Hospital Pharmaceutics, School of Pharmacy, Showa University, 1-5-8 Hatanodai, Shinagawa-ku, Tokyo, 142-8666 Japan; 4 Division of Pharmacy, Yamagata University Hospital, 2-2-2 Iida-nishi, Yamagata-shi, Yamagata, 990-9585 Japan; 5 Department of Pharmacy, Tohoku Medical and Pharmaceutical University Hospital, 1-12-1 Fukumuro, Miyagino-ku, Sendai, Miyagi 983-8512 Japan; 6 Department of Pharmacy, Miyagi Cancer Center, 47-1 Nodayama, Medeshimashiote, Natori, Miyagi 981-1293 Japan; 7 Department of Pharmacy, Kameda General Hospital, 929 Higashi-cho, Kamogawa-City, Chiba, 296-8602 Japan; 8 Department of Pharmaceutical Sciences, Tohoku University Hospital, 1-1, Seriyo-machi, Aoba-ku, Sendai, Miyagi 980-8575 Japan; 9 Division of Preventive Medicine and Epidemiology, Tohoku Medical Megabank Organization, 2-1, Seiryo-machi, Aoba-ku, Sendai, Miyagi 980-8573 Japan

**Keywords:** Additional reimbursement for infection prevention, Hospital pharmacist, Infection control, Japanese society of hospital pharmacists

## Abstract

**Background:**

Hospitals in Japan established the healthcare delivery system from FY 2018 to 2021 by acquiring an additional reimbursement for infection prevention (ARIP) of category 1 or 2. However, research on outcomes of ARIP applications related to the practice of hospital pharmacists is scarce.

**Methods:**

This study assessed the activities performed by hospital pharmacists in hospitals with 100 to 299 beds, using ARIP acquirement as an indicator, using data from an annual questionnaire survey conducted in 2020 by the Japanese Society of Hospital Pharmacists on the status of hospital pharmacy departments. Out of the survey items, this study used those related to hospital functions, number of beds, number of pharmacists, whether the hospital is included in the diagnosis procedure combination (DPC) system, average length of stay, and nature of work being performed in the analysis. The relationship between the number of beds per pharmacist and state of implementation of pharmacist services or the average length of hospital stay was considered uncorrelated when the absolute value of the correlation coefficient was within 0–0.2, whereas the relationship was considered to have a weak, moderate, or strong correlation when the absolute value ranged at 0.2–0.4, 0.4–0.7, or 0.7–1, respectively.

**Results:**

Responses were received from 3612 (recovery rate: 43.6%) hospitals. Of these, 210 hospitals meeting the criteria for ARIP 1 with 100–299 beds, and 245 hospitals meeting the criteria for ARIP 2 with 100–299 beds, were included in our analysis. There was a significant difference in the number of pharmacists, with a larger number in ARIP 1 hospitals. For the pharmacist services, significant differences were observed, with a more frequency in ARIP 1 hospitals in pharmaceutical management and guidance to pre-hospitalization patients, sterile drug processing of injection drugs and therapeutic drug monitoring. In DPC hospitals with ARIP 1 (173 hospitals) and 2 (105 hospitals), the average number of beds per pharmacist was 21.7 and 24.7, respectively, while the average length of stay was 14.3 and 15.4 d, respectively. Additionally, a weak negative correlation was observed between the number of pharmacist services with “Fairly well” or “Often” and the number of beds per pharmacist for both ARIP 1 (*R* = -0.207) and ARIP 2 (*R* = -0.279) DPC hospitals. Furthermore, a weak correlation (*R* = 0.322) between the average number of beds per pharmacist and the average length of hospital stay was observed for ARIP 2 hospitals.

**Conclusions:**

Our results suggest that lower beds per pharmacist might lead to improved pharmacist services in 100–299 beds DPC hospitals with ARIP 1 or 2. The promotion of proactive efforts in hospital pharmacist services and fewer beds per pharmacist may relate to shorter hospital stays especially in small and medium-sized hospitals with ARIP 2 when ARIP acquisition was used as an indicator. These findings may help to accelerate the involvement of hospital pharmacists in infection control in the future.

## Background

The importance of pharmacist involvement in infection control is increasing due to developments in medical care and multidrug-resistant bacterial infections [1]. In Japan, the FY 2018 revision to reimbursements for medical services established additional reimbursements for antimicrobial stewardship in addition to the existing additional reimbursement for infection prevention (ARIP). Consequently, the importance of preventative actions against infections by medical personnel, including pharmacists in hospitals, was recognized to a greater extent. The development by hospital pharmacists of systems using antimicrobials has reported to reduce the amount of antimicrobials used, drug costs, and the hospital days for patients receiving antimicrobial treatment [2]. An uncontrolled Before–After study found that antimicrobial therapy through pharmacist-led interventions improved clinical outcomes, including reduced incidences of hospital-acquired *Clostridioides difficile* infection and bacteremia-associated 30-d mortality rates [3]. Similar efforts were successful in relatively small hospitals. Collaboration among pharmacists conducting prescription audits, clinical pharmacists, and antimicrobial stewardship teams has also been reported to reduce the recurrence rates of infections after 30 d [4]. Therefore, pharmacists’ involvement in infection control is important, clinically and economically.

Several hospitals in Japan, between 2018 and 2021, established systems by acquiring either ARIP category 1 or 2 (ARIP 1 or 2), depending on the infection control practices and hospital size. The development, assessment and monitoring of pharmacist services at hospitals with ARIP 1 and ARIP 2 may increase the future involvement of hospital pharmacists in infection control. However, despite reports on outcome evaluation of antimicrobial stewardship programs [2, 4–6], studies examining outcomes of ARIP adoption in hospitals are lacking. Most reports on pharmacists’ efforts and outcomes of the appropriate use of antimicrobial agents are individual reports from large hospitals and intensive care units [7, 8], and comparative studies focusing on obtaining ARIP both on a national scale and in relatively small and medium-sized hospitals are scarce.

The Japanese Society of Hospital Pharmacists (JSHP), the largest professional organization for hospital pharmacists in Japan, conducts and publishes a nationwide survey on hospital pharmacist operations every year. This is a large-scale questionnaire-based survey open to all hospitals in Japan and is designed to assess the status of hospital pharmacy departments. In the current study, we utilized data from this survey to clarify the differences in pharmacy services provided by hospital pharmacists, especially in relatively small and medium-sized hospitals with 100 to 299 beds, using ARIP acquirement as an indicator.

## Methods

### Data collection

We used the results of a questionnaire survey conducted in 2020 by the JSHP on the status of hospital pharmacy departments in this study. No restrictions exist on the number of hospital beds for ARIP 1, but for ARIP 2, the number of general hospital beds must be less than 300. Additionally, few hospitals with a bed number less than 100 obtained ARIP. Therefore, we analyzed data for hospitals, with either ARIP 1 or ARIP 2, consisting of only 100 to 299 general hospital beds, to improve the reliability of this study, which implies that the current analysis partially includes hospitals that are not general hospitals, such as care-mix hospitals with only general hospital beds. The requirements for ARIP 1 and ARIP 2 are shown in Table 1. For both ARIP 1 and 2 hospitals, the pharmacist must be a member of the infection control team (ICT), and a permission or notification system of specific antimicrobial agents (e.g., broad-spectrum antimicrobial or anti-MRSA agents) is operated. The JSHP questionnaire consists of 43 items, which include facility functions and overview and pharmacist and pharmacy department overview, and each item contains several related questions. We used those related to hospital functions, number of beds, number of pharmacists, the inclusion of the hospital in the diagnosis procedure combination (DPC) system, average length of patients’ stay, and the nature of work being performed. The facilities with an average length of stay of ±5 standard deviations or more, less than 20 beds, or less than one pharmacist were excluded.

**Table 1:** Additional reimbursement for infection prevention

Categories	Additional reimbursement for infection prevention 1 (ARIP 1)	Additional reimbursement for infection prevention 2 (ARIP 2)
Outline	Calculate on the first hospitalized day for inpatients in an insured medical institution, which has an infection control team (ICT) in the hospital to prevent nosocomial infections by monitoring the infection situation in the hospital, appropriate use of antimicrobial agents, and preventing infection in the hospital staff.	
Medical remuneration points	390 points (1 point = JPY 10)	90 points (1 point = JPY 10)
Hospital bed size	No conditions	The standard number of general hospital beds is 300 or less
Infection Control Team Members	• Full-time physician (with at least 3 years of experience in infection control) • Full-time nurse (with at least 5 years of experience in infection control and completion of the training program) • Full-time pharmacist (at least 3 years of working experience in a hospital) • Full-time clinical technologist (with at least 3 years of working experience in a hospital)	• Full-time physician (with at least 3 years of experience in infection control) • Full-time nurse (with at least 5 years of experience in infection control) • Full-time pharmacist (at least 3 years of working experience in a hospital) • Full-time clinical technologist (with at least 3 years of working experience in a hospital)
	One physician or nurse must be working in the ICT for at least 80% of the working hours, and other professionals must be working in the ICT for at least 50% of the working hours.	All professions must be working in the ICT at least 50% of the working hours.
Standard operating procedure	The ICT must prepare standard operating procedures, which include standard precautions, tailored to the actual conditions of the facility, and precautions by route of infection, based on the latest evidence and tailored to the actual situation at each facility.	
Operations	• Permission or notification system of specific antimicrobial agents (e.g., broad-spectrum antimicrobial or anti-MRSA agents) • Regular in-hospital round • Participation in regional and national surveillance	• Permission or notification system of specific antimicrobial agents (e.g., broad-spectrum antimicrobial or anti-MRSA agents) • Regular in-hospital round
Education and training	Regular training on nosocomial infection control must be conducted.	
Cooperation with other medical institutions	• Holding of conferences, at least four times a year, with the hospitals of additional reimbursement for infection prevention category 2 • Reception of consultations, when necessary, from hospitals of additional reimbursement for infection prevention category 2 on nosocomial infection control	• Participant in conferences, at least four times a year, held by the hospitals of additional reimbursement for infection prevention category 1

### Statistical analysis

Statistical analyses were performed using the JMP® Pro 16 (SAS Institute), IBM SPSS Statistics 25 (Japan IBM, Ltd.), and Excel® for Mac (Microsoft). The Mann–Whitney *U* test and Fisher’s exact probability test were used for continuous and categorical variables, respectively. For multiple linear regression analysis, to evaluate the relationship between hospital function or the number of pharmacists and the average length of hospital stay, the objective variable was an average length of stay, and the explanatory variables were ARIP category (0: ARIP 1, 1: ARIP 2), DPC classification (0: DPC hospital, 1: non-DPC hospital), number of beds, number of pharmacists, and number of beds per pharmacist. *P* < 0.05 was considered statistically significant, and the value of the variance inflation factor (VIF) over 10 was considered to being multicollinearity. The relationship between the number of beds per pharmacist and state of implementation of pharmacist services or the average length of hospital stay was considered uncorrelated when the absolute value of the correlation coefficient was within 0–0.2, whereas the relationship was considered to have a weak, moderate, or strong correlation when the absolute value ranged at 0.2–0.4, 0.4–0.7, or 0.7–1, respectively. However, in the analysis of the relationship between the number of beds per pharmacist and state of implementation of pharmacist services, six ARIP 1 hospitals and one ARIP 2 hospital that answered “NA” for all pharmacist services were excluded from this analysis.

### Ethical approval

This study was approved by the Ethical Review Committee of Tohoku University Tohoku Medical Megabank Organization (Approval No. 2021–4-074).

## Results

### Characteristics of hospitals with ARIP 1 and ARIP 2

**Fig. 1:**
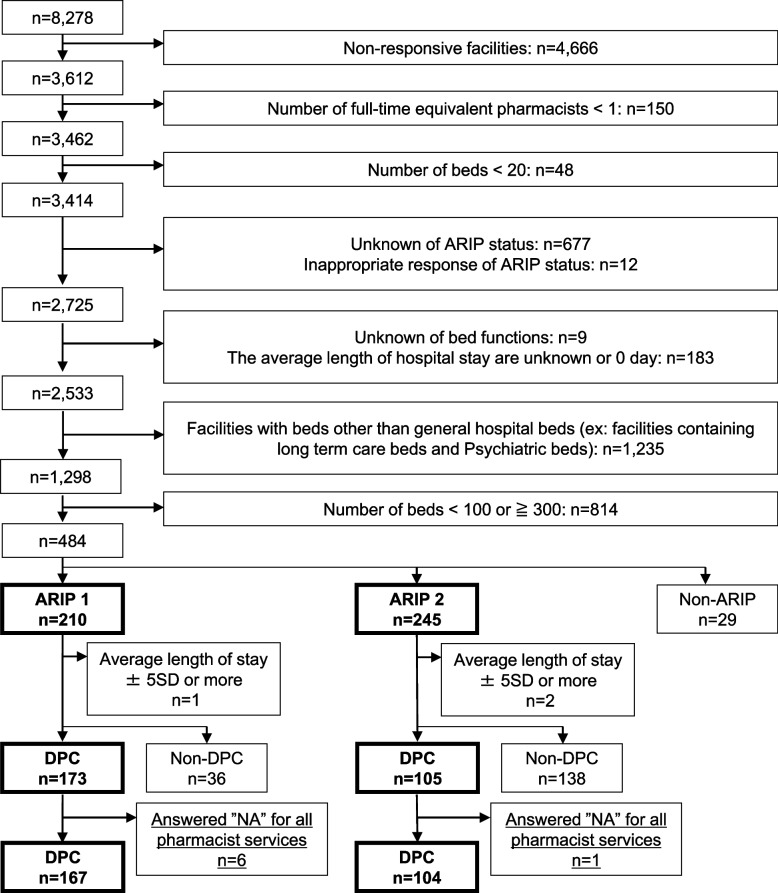
Extraction criteria and flow diagram of selected hospitals for analysis

Of the 8278 hospitals that were part of the annual survey, responses were received from 3612 hospitals (recovery rate: 43.6%). Out of these 3612 hospitals, 210 hospitals meeting the criteria for ARIP 1 with 100–299 beds, and 245 hospitals meeting the criteria for ARIP 2 with 100–299 beds, were included in our analysis (Fig. 1). Among the pharmacist services, significant differences were observed between ARIP 1 and 2 hospitals in “sterile dispensing services for inpatients,” “sterile dispensing services for outpatients,” and “therapeutic drug monitoring (TDM) services.” ARIP 1 hospitals were more frequently graded as “Fairly well (more than 80%)” and “Often (more than 50%)” than ARIP 2 hospitals. ARIP 1 hospitals were more frequently assessed as “Fairly well” or “Often” for working on education/research (including trainee guidance) (Table 2).

**Table 2:** Comparison of hospital functions and pharmacist operations (all facilities)

Categories of additional reimbursement for infection prevention		ARIP 1 (*n* = 210)	ARIP 2 (*n* = 245)	*P*-value
Number of pharmacists	1	0 (0.0%)	0 (0.0%)	< 0.05
	2–4	7 (3.3%)	81 (33.1%)	
	5–9	89 (42.4%)	117 (47.8%)	
	10–19	99 (47.1%)	42 (17.1%)	
	≥20	15 (7.1%)	5 (2.0%)	
Introduction of the DPC system	DPC hospital	174 (82.9%)	105 (42.9%)	< 0.05
	DPC preparatory hospital	5 (2.4%)	20 (8.2%)	
	Non-DPC hospital	30 (14.3%)	115 (46.9%)	
	NA	1 (0.5%)	5 (2.0%)	
Types of medical institutions	General hospitals	199 (94.8%)	217 (88.6%)	0.0518
	Care-mix^a^	10 (4.8%)	23 (9.4%)	
	NA	1 (0.5%)	5 (2.0%)	
Pharmaceutical management and guidance to inpatients	Fairly well	118 (56.2%)	131 (53.5%)	0.1700
	Often	59 (28.1%)	65 (26.5%)	
	Sometimes	27 (12.9%)	38 (15.5%)	
	Not implemented	0 (0.0%)	6 (2.4%)	
	NA	6 (2.9%)	5 (2.0%)	
Pharmaceutical management and guidance to pre-hospitalization patients	Fairly well	61 (29.0%)	88 (35.9%)	< 0.05
	Often	39 (18.6%)	32 (13.1%)	
	Sometimes	62 (29.5%)	54 (22.0%)	
	Not implemented	41 (19.5%)	66 (26.9%)	
	NA	7 (3.3%)	5 (2.0%)	
Pharmaceutical management and guidance to outpatients	Fairly well	28 (13.3%)	23 (9.4%)	< 0.05
	Often	28 (13.3%)	19 (7.8%)	
	Sometimes	103 (49.0%)	117 (47.8%)	
	Not implemented	42 (20.0%)	80 (32.7%)	
	NA	9 (4.3%)	6 (2.4%)	
Dispensing for inpatients (internal and external drugs)	Fairly well	168 (80.0%)	203 (82.9%)	0.0715
	Often	22 (10.5%)	33 (13.5%)	
	Sometimes	10 (4.8%)	5 (2.0%)	
	NA	10 (4.8%)	4 (1.6%)	
Dispensing for inpatients (injection drugs)	Fairly well	154 (73.3%)	183 (74.7%)	0.4580
	Often	35 (16.7%)	32 (13.1%)	
	Sometimes	13 (6.2%)	19 (7.8%)	
	Not implemented	0 (0.0%)	3 (1.2%)	
	NA	8 (3.8%)	8 (3.3%)	
Dispensing and management guidance for outpatients (internal, external, and injection drugs)	Fairly well	49 (23.3%)	63 (25.7%)	0.6859
	Often	28 (13.3%)	21 (8.6%)	
	Sometimes	119 (56.7%)	134 (54.7%)	
	Not implemented	1 (0.5%)	19 (7.8%)	
	NA	13 (6.2%)	8 (3.3%)	
Sterile drug processing of injection drugs for inpatients	Fairly well	67 (31.9%)	50 (20.4%)	< 0.05
	Often	51 (24.3%)	34 (13.9%)	
	Sometimes	71 (33.8%)	79 (32.2%)	
	Not implemented	13 (6.2%)	76 (31.0%)	
	NA	8 (3.8%)	6 (2.4%)	
Sterile drug processing of injection drugs for outpatients	Fairly well	63 (30.0%)	39 (15.9%)	< 0.05
	Often	37 (17.6%)	21 (8.6%)	
	Sometimes	65 (31.0%)	76 (31.0%)	
	Not implemented	37 (17.6%)	102 (41.6%)	
	NA	8 (3.8%)	7 (2.9%)	
Drug information management	Fairly well	78 (37.1%)	87 (35.5%)	0.6859
	Often	66 (31.4%)	88 (35.9%)	
	Sometimes	57 (27.1%)	63 (25.7%)	
	NA	9 (4.3%)	7 (2.9%)	
Therapeutic drug monitoring	Fairly well	31 (14.8%)	22 (9.0%)	< 0.05
	Often	47 (22.4%)	27 (11.0%)	
	Sometimes	106 (50.5%)	128 (52.2%)	
	Not implemented	18 (8.6%)	61 (24.9%)	
	NA	8 (3.8%)	7 (2.9%)	
Participation in cross-hospital medical teams (e.g., infection control team, antimicrobial stewardship team, and nutrition support team)	Fairly well	82 (39.0%)	73 (29.8%)	< 0.05
	Often	63 (30.0%)	79 (32.2%)	
	Sometimes	51 (24.3%)	79 (32.2%)	
	Not implemented	0 (0.0%)	4 (1.6%)	
	NA	14 (6.7%)	10 (4.1%)	
Education (includes pharmacy student education) and Research	Fairly well	40 (19.0%)	22 (9.0%)	< 0.05
	Often	40 (19.0%)	32 (13.1%)	
	Sometimes	77 (36.7%)	65 (26.5%)	
	Not implemented	45 (21.4%)	113 (46.1%)	
	NA	8 (3.8%)	13 (5.3%)	
Percentage of out-of-hospital prescriptions issued	≥80%	175 (83.3%)	182 (74.3%)	0.1369
	60% to less than 80%	6 (2.9%)	6 (2.4%)	
	40% to less than 60%	2 (1.0%)	3 (1.2%)	
	< 40%	22 (10.5%)	45 (18.4%)	
	NA	5 (2.4%)	9 (3.7%)	

*ARIP* additional reimbursement for infection prevention, *DPC* Diagnosis Procedure Combination, *NA* no answer

^a^Hospitals with a combination of general and convalescent or psychiatric beds

**Fig. 2:**
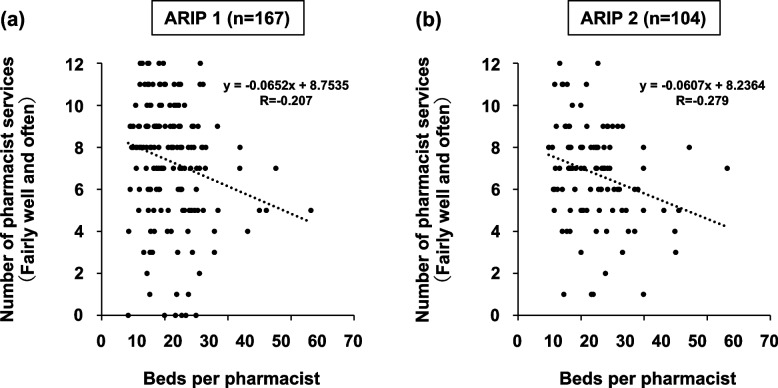
Relationship between state of implementation of pharmacist services and number of beds per pharmacist (DPC hospitals). **a** Additional reimbursement for infection prevention category 1 and DPC hospitals. **b** Additional reimbursement for infection prevention category 2 and DPC hospitals

To understand the characteristics of the subject facility, multiple linear regression analysis was performed. The objective variable was the average length of stay Median value, and the explanatory variables were ARIP category, DPC classification, number of beds, and number of pharmacists. DPC classification (B = − 28.234, β = − 0.160, *P* = 0.003, VIF = 1.417), number of beds (B = 0.321, β = 0.197, *P* = 0.001, VIF = 1.730), and number of pharmacists (B = − 2.734, β = 0.161, *P* = 0.007, VIF = 1.683) were extracted as factors affecting the average length of hospital stay. Thus, to better align backgrounds, a comparison of hospitals subject to DPC for ARIP 1 and 2 (ARIP 1: 173 hospitals; ARIP 2: 105 hospitals) was conducted. The results revealed a significant difference in the number of pharmacists, with a larger number in ARIP 1 hospitals. Among the pharmacist services, significant differences were observed in “Pharmaceutical management and guidance to pre-hospitalization patients,” “Dispensing and management guidance for outpatients,” “sterile preparation and processing services for inpatients,” and “sterile preparation and processing services for outpatients,” with a larger number in ARIP 1 hospitals. ARIP 1 hospitals generally more participation in educational and research activities. No difference was observed in the rate of issuance of outpatient prescriptions between ARIP 1 and 2 hospitals (Table 3). Additionally, a weak negative correlation was observed between the number of pharmacist services with “Fairly well” or “Often” and the number of beds per pharmacist for both ARIP 1 (*R* = -0.207) and ARIP 2 (*R* = -0.279) DPC hospitals (Fig. 2).

**Table 3:** Comparison of hospital functions and pharmacist operations (DPC hospitals)

Categories of additional reimbursement for infection prevention		ARIP 1 (*n* = 173)	ARIP 2 (*n* = 105)	*P*-value
Number of pharmacists	1	0 (0.0%)	0 (0.0%)	< 0.05
	2–4	4 (2.3%)	14 (13.3%)	
	5–9	65 (37.4%)	55 (52.4%)	
	10–19	93 (54.0%)	34 (32.4%)	
	≥20	11 (6.3%)	2 (1.9%)	
Types of medical institutions	General hospitals	165 (95.4%)	92 (87.6%)	< 0.05
	Care-mix^a^	7 (4.0%)	11 (10.5%)	
	NA	1 (0.6%)	2 (1.9%)	
average number of beds per pharmacist	Mean value (95% CI)	21.7 (20.4–23.1)	24.7 (22.6–26.8)	< 0.05
Average length of hospital stay	Median value (min-max)	14.3 (12.3–17.0)	15.4 (13.7–24.0)	< 0.01
Pharmaceutical management and guidance to inpatients	Fairly well	99 (57.2%)	69 (65.7%)	0.4150
	Often	47 (27.2%)	24 (22.7%)	
	Sometimes	21 (12.1%)	9 (8.6%)	
	Not implemented	0 (0.0%)	1 (1.0%)	
	NA	6 (3.5%)	2 (1.9%)	
Pharmaceutical management and guidance to pre-hospitalization patients	Fairly well	48 (27.8%)	42 (40.0%)	< 0.05
	Often	34 (19.7%)	9 (8.6%)	
	Sometimes	52 (30.0%)	29 (27.6%)	
	Not implemented	32 (18.5%)	24 (22.9%)	
	NA	7 (4.0%)	1 (1.0%)	
Pharmaceutical management and guidance to outpatients	Fairly well	27 (15.6%)	8 (7.6%)	0.0709
	Often	26 (15.0%)	10 (9.5%)	
	Sometimes	85 (49.1%)	57 (54.3%)	
	Not implemented	26 (15.0%)	26 (24.8%)	
	NA	9 (5.2%)	4 (3.8%)	
Dispensing for inpatients (internal and external drugs)	Fairly well	136 (78.6%)	90 (85.7%)	0.3422
	Often	18 (10.4%)	10 (9.5%)	
	Sometimes	9 (5.2%)	3 (2.9%)	
	NA	10 (5.8%)	2 (1.9%)	
Dispensing for inpatients (injection drugs)	Fairly well	124 (71.7%)	79 (75.2%)	0.3464
	Often	30 (17.3%)	13 (12.4%)	
	Sometimes	11 (6.3%)	6 (5.7%)	
	Not implemented	0 (0.0%)	3 (2.9%)	
	NA	8 (4.6%)	4 (3.8%)	
Dispensing and management guidance for outpatients (internal, external, and injection drugs)	Fairly well	41 (23.7%)	23 (21.9%)	< 0.05
	Often	26 (15.0%)	12 (11.4%)	
	Sometimes	96 (55.5%)	60 (57.1%)	
	Not implemented	0 (0.0%)	6 (5.7%)	
	NA	10 (5.8%)	4 (3.8%)	
Sterile drug processing of injection drugs for inpatients	Fairly well	56 (32.3%)	32 (30.5%)	< 0.05
	Often	44 (25.4%)	19 (18.1%)	
	Sometimes	61 (35.3%)	32 (30.5%)	
	Not implemented	5 (2.9%)	20 (19.1%)	
	NA	7 (4.0%)	4 (3.8%)	
Sterile drug processing of injection drugs for outpatients	Fairly well	55 (31.8%)	30 (28.6%)	< 0.05
	Often	34 (19.7%)	12 (11.4%)	
	Sometimes	54 (31.2%)	35 (33.3%)	
	Not implemented	22 (12.7%)	26 (24.8%)	
	NA	8 (4.6%)	2 (1.9%)	
Drug information management	Fairly well	69 (39.9%)	46 (43.8%)	0.2289
	Often	51 (29.5%)	38 (36.2%)	
	Sometimes	44 (25.4%)	19 (18.1%)	
	NA	9 (5.2%)	2 (1.9%)	
Therapeutic drug monitoring	Fairly well	29 (16.8%)	14 (13.3%)	0.0744
	Often	44 (25.4%)	18 (17.1%)	
	Sometimes	79 (45.7%)	54 (51.4%)	
	Not implemented	13 (7.5%)	17 (16.2%)	
	NA	8 (4.6%)	2 (1.9%)	
Participation in cross-hospital medical teams (e.g., infection control team, antimicrobial stewardship team, and nutrition support team)	Fairly well	68 (39.4%)	40 (38.1%)	0.6093
	Often	51 (29.5%)	32 (30.5%)	
	Sometimes	40 (23.1%)	27 (25.7%)	
	Not implemented	0 (0.0%)	1 (1.0%)	
	NA	14 (8.1%)	5 (4.8%)	
Education (includes pharmacy student education) and Research	Fairly well	34 (19.7%)	13 (12.4%)	< 0.05
	Often	35 (20.2%)	18 (17.1%)	
	Sometimes	62 (35.8%)	30 (28.6%)	
	Not implemented	34 (19.7%)	39 (37.1%)	
	NA	8 (4.6%)	5 (4.8%)	
Percentage of out-of-hospital prescriptions issued	≥80%	145 (83.8%)	86 (81.9%)	0.9279
	60% to less than 80%	5 (2.9%)	4 (3.8%)	
	40% to less than 60%	1 (0.6%)	0 (0.0%)	
	< 40%	17 (9.8%)	13 (12.3%)	
	NA	5 (2.9%)	2 (1.9%)	

*ARIP* additional reimbursement for infection prevention, *DPC* Diagnosis Procedure Combination, *NA* no answer

^a^Hospitals with a combination of general and convalescent or psychiatric beds

### Relationship between bed per pharmacist and the average length of stay

**Fig. 3:**
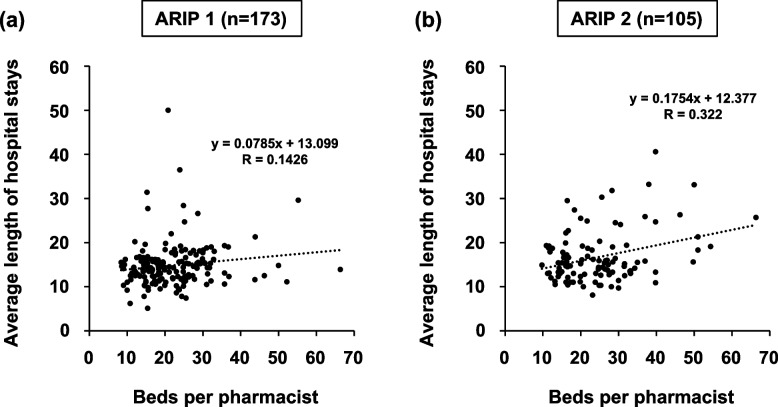
Relationship between the average length of hospital stay and number of beds per pharmacist (DPC hospitals). **a** Additional reimbursement for infection prevention category 1 and DPC hospitals. **b** Additional reimbursement for infection prevention category 2 and DPC hospitals

In DPC hospitals with ARIP 1 and 2, the average number of beds per pharmacist (95% CI) was 21.7 (20.4–23.1) and 24.7 (22.6–26.8) (*P* < 0.05), and the average length of stay (d) (median value: min–max) was 14.3 (12.3–17.0) and 15.4 (13.7–24.0) (*P* < 0.01), respectively (Table 3). No correlation between the average number of beds per pharmacist and the average length of hospital stay was observed for ARIP 1, but however, a weak correlation (*R* = 0.322) was observed for ARIP 2 (Fig. 3). To confirm this result and identify the effect of other factors, multiple linear regression analysis was performed. As a result, though no factors affecting the average length of stay were extracted in ARIP 1 hospitals (number of beds [B = 0.001, β = 0.07, *P* = 0.950, VIF = 1.991], number of pharmacists [B = − 0.158, β = − 0.161, *P* = 0.307, VIF = 4.334], and beds per pharmacist [B = 0.013, β = 0.023, *P* = 0.875, VIF = 3.665]), beds per pharmacist was extracted as an affecting factor on the average length of stay was extracted in ARIP 2 hospitals (number of beds [B = − 0.033, β = − 0.237, *P* = 0.092, VIF = 2.254], number of pharmacists [B = 0.383, β = 0.282, *P* = 0.173, VIF = 4.881], and beds per pharmacist [B = 0.284, β = 0.521, *P* = 0.003, VIF = 3.512]).

## Discussion

To our knowledge, this is the first study to clarify the status of hospital pharmacist operations at hospitals with 100 to 299 general hospital beds, using data from the 2020 survey by the JSHP.

Our findings revealed that hospitals with ARIP 1 had more pharmacists and DPC systems than those with ARIP 2. Pharmacists generally provided more sterile processing and TDM services for both inpatients and outpatients in hospitals with ARIP 1 than those with ARIP 2. No difference was observed in the rate of outpatient prescriptions issued in ARIP 1 and 2 hospitals. These results may reflect the effort that obtained ARIP 1 with more advanced requirements than ARIP2 on multiple indicators (Table 1) even though ARIP 2 can be obtained for hospitals with 100 to 299 beds. Based on the analysis comparing DPC hospitals with 100 to 299 beds, the number of beds per pharmacist was generally less, and the average length of stay was shorter in hospitals with ARIP 1 than those with ARIP 2. Previous research report increases in the consumption of infection control supplies and promotion of infection control activities by the transfer from ARIP 2 to ARIP 1, along with a decrease in the number of patients with MRSA infection and the incidence rate of MRSA infections [9]. Therefore, although an increase in expenditure for countermeasure maintenance items is needed for ARIP 2 hospitals to obtain ARIP 1, the reduction of healthcare-associated infections may be beneficial in reducing the average length of stay and the resulting inpatient medical costs. DPC hospitals were selected as a background for our analysis because it was an important factor in the multiple regression analysis conducted. Our results showed that 86.9% of the hospitals with ARIP 1 had an antimicrobial stewardship team compared with 21.9% of those with ARIP 2 (data not shown). These results hint that the presence of an antimicrobial stewardship team might reduce the average length of hospital stay. However, establishing an antimicrobial stewardship team is also one of the actions performed toward infection control by ARIP hospitals.

For both ARIP 1 and 2 hospitals, a weak negative correlation was observed between the number of pharmacist services with “Fairly well” or “Often” and the number of beds per pharmacist. Thus, lower beds per pharmacist might lead to improved pharmacist services regardless of the type of ARIP acquirement in DPC hospitals with 100–299 beds. In contrast, a weak correlation between the average number of hospital beds per pharmacist and the average length of hospital stay was observed for only ARIP 2 hospitals. Although the VIF value was over 3, these results were also supported by the results of the multiple regression analysis of this study. As shown in Table 3, ARIP 1 hospitals have already provided a greater extent of pharmacist services, particularly for inpatients. Therefore, the average length of stay in ARIP 1 hospitals was likely to be unrelated to the average number of beds per pharmacist. In contrast, for ARIP 2 hospitals that realize the difficulty in obtaining ARIP 1, decreasing the number of beds per pharmacist and increasing their activities, such as pharmaceutical management and guidance to pre-hospitalization patients, sterile drug processing and TDM services, may contribute to shorter hospital stays. A survey involving 1358 hospitals in Japan reported a strong positive correlation between the number of full-time equivalent physicians and pharmacists and the number of items implemented in antimicrobial stewardship programs [10]. Furthermore, the establishment of antimicrobial stewardship programs by doctors and pharmacists is reported to contribute to shorter hospital stays [2]. Thus, lower beds per pharmacist may also contribute to shorter hospital stays through increased human resources engaging in antimicrobial stewardship programs. In this study, with the average length of hospital stay as the outcome, the correlation with the average number of beds per pharmacist remained weak (*R* = 0.322), even in ARIP 2 hospitals. One reason could be that infection control is often achieved through an integrated approach involving different professionals [11]. However, various pharmacist-led initiatives have been reported to promote the use of antimicrobial agents, improve achievement rate of effective blood concentration range in vancomycin (VCM) therapy [12], reducing the rate of MRSA in intensive care units and in antimicrobial allergic reactions [13–16]. In addition, a retrospective study has shown that the survival rate at 30 days after starting VCM therapy with pharmacist-led VCM initial dose planning was higher than that of non-intervention groups in MRSA bacteremia patients [17]. Drugs used for the treatment of infectious diseases are also the most common drugs for which adverse drug reactions are avoided through pharmacological intervention by hospital pharmacists [18]. Thus, the efforts to increase the involvement of pharmacists in infection control are vital for improving its safety and efficacy.

One of the limitations of this study is that it is based on a survey and has an overall response rate of 43.6%. Therefore, this study includes only those hospitals that responded to the survey, and this sample may not reflect the whole population. However, since a survey of this scale has not been conducted in Japan to date, we believe that this study is the first to identify the role of hospital pharmacists, particularly in relatively small and medium-sized institutions with 100 to 299 beds. Moreover, this study included non-general hospitals with only general hospital beds (care-mix hospitals: 7.3%, 33/455; NA: 1.3%, 6/455) under the hospital category. We were unable to examine the effect of hospitals with beds dedicated to infectious diseases (8.1%, 37/455) or tuberculosis (4.8%, 22/455), the number of antimicrobials used, the incidence of antimicrobial resistance in each hospital, activities of infection control teams or another health-professions, and regional characteristics. As this study was based on secondary use of the JSHP’s questionnaire results, although the explanatory variables were selected from the results of preliminary single regression analysis, there were limitations on the items that could be used for multiple regression analysis. Thus, the relevance of factors not examined in the multiple regression analysis (e.g., the number of pharmaceutical interventions related to infection control by pharmacists) is open to further research. Finally, the ARIP is not an additional fee for pharmacists, but for the system and activities of the ICT is also should be noted. Nevertheless, the results of this study provide useful information on the characteristics of pharmacist services in hospitals with only 100–299 general hospital beds, using the ARIP as an indicator.

## Conclusions

This study based on the data from the 2020 annual Hospital Pharmacy Survey. Our results suggest that lower beds per pharmacist might lead to improved pharmacist services in 100–299 beds DPC hospitals with ARIP 1 or 2. The promotion of proactive efforts in hospital pharmacist services and fewer beds per pharmacist may relate to shorter hospital stays especially in small and medium-sized DPC hospitals with ARIP 2 when ARIP acquisition was used as an indicator. These findings may help to accelerate the involvement of hospital pharmacists in infection control in the future.

## Data Availability

Not applicable.

## Abbreviations

ARIP: Additional reimbursement for infection prevention
DPC: Diagnosis procedure combination
ICT: Infection control team
JSHP: Japanese Society of Hospital Pharmacists
MRSA: Methicillin-resistant *Staphylococcus aureus*
TDM: Therapeutic drug monitoring
VCM: Vancomycin
VIF: Variance inflation factor
